# The Medical Student’s Guide

**Published:** 1842-11-19

**Authors:** 


*The Medical Student's Guide.* By HEBER CHASE, M. D. J. G. Auner pp. 101.

A very useful little compendium, containing the fullest information respecting the schools, hospitals, and other medical institutions of Philadelphia. It will be found a serviceable vade mecum to students visiting this city.

